# Human central nervous system astrocytes support survival and activation of B cells: implications for MS pathogenesis

**DOI:** 10.1186/s12974-018-1136-2

**Published:** 2018-04-19

**Authors:** Hanane Touil, Antonia Kobert, Nathalie Lebeurrier, Aja Rieger, Philippe Saikali, Caroline Lambert, Lama Fawaz, Craig S. Moore, Alexandre Prat, Jennifer Gommerman, Jack P. Antel, Yasuto Itoyama, Ichiro Nakashima, Amit Bar-Or, for the Canadian B Cell Team in MS, A. Rezk, F. Jalili, L. Michel, N. Pikor, R. Li

**Affiliations:** 1 Neuroimmunology Unit, Montreal Neurological Institute, McGill University, 3801 University Street, Room 111, Montréal , QC H3A 2B3 Canada; 2 Department of Neurology and Center for NeuroInflammation and Experimental Therapeutics (CNET), Perelman School of Medicine, University of Pennsylvania, Philadelphia, PA USA; 3 Division of BioMedical Sciences, Faculty of Medicine, Memorial University of Newfoundland, St. John’s, NF Canada; 4 Université de Montréal Centre de Recherche du CHUM (CRCHUM) and Department of Neuroscience, Université de Montréal, 900 Saint Denis Street, Montreal, QC H2X 0A9 Canada; 5 Department of Immunology, Medical Sciences Building, University of Toronto, Toronto, ON M5S 1A8 Canada; 6 Department of Neurology, School of Medicine, Tohoku University, 1–1 Seiryo-machi, Aoba-ku, Sendai, Japan

**Keywords:** Multiple sclerosis, CNS-compartmentalized inflammation, Human B cells, Human astrocytes

## Abstract

**Background:**

The success of clinical trials of selective B cell depletion in patients with relapsing multiple sclerosis (MS) indicates B cells are important contributors to peripheral immune responses involved in the development of new relapses. Such B cell contribution to peripheral inflammation likely involves antibody-independent mechanisms. Of growing interest is the potential that B cells, within the MS central nervous system (CNS), may also contribute to the propagation of CNS-compartmentalized inflammation in progressive (non-relapsing) disease. B cells are known to persist in the inflamed MS CNS and are more recently described as concentrated in meningeal immune-cell aggregates, adjacent to the subpial cortical injury which has been associated with progressive disease. How B cells are fostered within the MS CNS and how they may contribute locally to the propagation of CNS-compartmentalized inflammation remain to be elucidated.

**Methods:**

We considered whether activated human astrocytes might contribute to B cell survival and function through soluble factors. B cells from healthy controls (HC) and untreated MS patients were exposed to primary human astrocytes that were either maintained under basal culture conditions (non-activated) or pre-activated with standard inflammatory signals. B cell exposure to astrocytes included direct co-culture, co-culture in transwells, or exposure to astrocyte-conditioned medium. Following the different exposures, B cell survival and expression of T cell co-stimulatory molecules were assessed by flow cytometry, as was the ability of differentially exposed B cells to induce activation of allogeneic T cells.

**Results:**

Secreted factors from both non-activated and activated human astrocytes robustly supported human B cell survival. Soluble products of pre-activated astrocytes also induced B cell upregulation of antigen-presenting cell machinery, and these B cells, in turn, were more efficient activators of T cells. Astrocyte-soluble factors could support survival and activation of B cell subsets implicated in MS, including memory B cells from patients with both relapsing and progressive forms of disease.

**Conclusions:**

Our findings point to a potential mechanism whereby activated astrocytes in the inflamed MS CNS not only promote a B cell fostering environment, but also actively support the ability of B cells to contribute to the propagation of CNS-compartmentalized inflammation, now thought to play key roles in progressive disease.

**Electronic supplementary material:**

The online version of this article (10.1186/s12974-018-1136-2) contains supplementary material, which is available to authorized users.

## Background

Studies of immune mechanisms that contribute to multiple sclerosis (MS) pathophysiology have traditionally highlighted processes of aberrant peripheral immune-cell activation and their subsequent trafficking into the central nervous system (CNS). This paradigm, commonly studied in animal models such as experimental autoimmune encephalomyelitis (EAE), continues to provide a useful framework for understanding as well as therapeutically targeting peripheral immune responses that underlie MS relapses. However, a major unmet clinical need in MS is represented by a relentless, progressive (non-relapsing) disease course, eventually experienced by most patients. The biology underlying such progression is less well understood but is now thought to reflect, at least in part, ongoing inflammation that is compartmentalized within the CNS. How such inflammation is maintained and how it is propagated within the CNS of patients are unknown.

Pathologic studies in recent years have highlighted the presence of immune cells (i.e., inflammation) within the meninges of patients with MS [1–3]. An important association has been identified between such meningeal inflammation and cortical injury, in particular, the subpial form of cortical pathology, which can be very extensive and is now thought to represent an important substrate of progressive disease [4, 5]. This subpial cortical pathology is characterized by oligodendrocyte injury, demyelination, and astrogliosis, together with a graded degree of neuronal loss and microglial activation [1, 3]. The extent of neuronal loss and microglial activation in subpial lesions is reportedly greatest in superficial layer I of the cortex (immediately underlying the pia-meninges and cerebrospinal fluid (CSF)), with lesser abnormalities seen through the deeper cortical layers. An attractive hypothesis currently being pursued in the field is that progressive subpial cortical injury may reflect the consequences of one or more soluble factors secreted by immune cells fostered in the meninges.

A spectrum of meningeal inflammation has been described in MS ranging from relatively scattered immune cells to considerably more organized immune-cell collections, some of which recapitulate features of tertiary lymphoid structures. While variable degrees of T cells and myeloid cells have been described across this spectrum, a commonly highlighted feature of meningeal inflammation in MS is the presence of B cells and plasma cells, with several studies describing meningeal immune-cell collections that are “B-cell rich” [1, 2, 6, 7]. B cells of MS patients are known to be abnormally pro-inflammatory, and their soluble products in vitro can be toxic to oligodendrocytes and neurons, fueling an interest in the potential contribution of B cells to CNS-compartmentalized inflammation and progressive CNS injury in MS [8, 9].

In this regard, the inflamed MS CNS appears to be a B cell fostering environment, as evidenced by the remarkable persistence and clonal uniformity of intrathecal immunoglobulin (Ig), and of serially sampled B cells and plasma cells [10]. However, the mechanisms that may support B cell survival within the MS CNS and the implications for propagation of local disease processes have not been fully elucidated. Here, we assessed whether and how soluble factors released by human astrocytes may impact survival and activation of B cell responses that could, in turn, contribute to propagating CNS-compartmentalized inflammatory responses.

## Methods

### Participants

Healthy controls (HC) were recruited among members of the McGill University community. Well-characterized patients with confirmed MS (Additional file 1: Table S1) were recruited at the MS Clinic of the Montreal Neurological Institute and Hospital (MNI/H). All participants provided informed consent as part of a protocol approved by the university’s internal review board (IRB). All MS patients were untreated at the time of blood draw, had not received any immune-modulating treatments within at least 6 months prior to sampling, and had not been treated with steroids within at least 30 days prior to sampling.

### B cell and B cell subset isolation

B cells were isolated from fresh antecubital venous blood, as previously described [11]. Briefly, peripheral blood mononuclear cells (PBMC) were isolated from 100 to 120 ml venous blood of untreated MS patients and healthy volunteers using standard density-gradient centrifugation on Ficoll-Paque (Pharmacia Biotech). Magnetic beads (MACS, Miltenyi Biotec) were used according to the manufacturer’s instructions to isolate CD19^+^ B cells by positive selection, and their purity was confirmed by flow cytometry (routinely > 98% pure). B cells were then washed and resuspended in serum-free X-Vivo 10 medium (Lonza, Walkersville, MD). For experiments with B cell subsets, total B cells were initially sorted from PBMC by CD19+ MACS separation and then stained for CD20^+^ (2H7), CD27^+^ (M-T271), IgD (IA6-2), CD24 (ML5), and CD38 (HIT2), all from BD Bioscience. The total B cells were subsequently sorted (using a BD LSRFortessa, BD Bioscience) into transitional (CD20^+^CD24^+^CD38^+^), naive (CD20^+^CD27^−^IgD^+^), or memory (CD20^+^CD27^+^IgD^−/+^) B cell subsets with routine purity confirmation (typically > 93%).

### Astrocyte isolation and culture

Human fetal astrocytes (HFA) were isolated as previously described [12]. In brief, fetal CNS tissue age 17–22 weeks of gestation, obtained from the Albert Einstein College of Medicine Human Fetal Tissue Repository (Bronx, NY), was first dissociated using trypsin-EDTA (Invitrogen Life Technologies) and DNase I (Roche, Laval, QC), followed by mechanical dissociation. The cell suspension was then washed and seeded in complete Dulbecco’s modified Eagle’s medium (DMEM; containing 10% fetal calf serum (FCS), penicillin, streptomycin, l-glutamine, and glucose) into poly-l-lysine-coated flasks, at a concentration of 3–5 × 10^6^ cells/mL. To obtain near-pure astrocyte cultures, the HFA were further enriched in DMEM by at least three passages, starting 2 weeks post isolation. Astrocytes for our experiments, isolated from over 40 independent preparations, were used between passages 3 and 5 and > 90% pure, as determined by glial fibrillary acidic protein (GFAP, clone EPG724) immunostaining as previously described [13]. Purified astrocytes were then washed twice in phosphate-buffered saline (PBS) and plated in complete DMEM at a density of 0.1 × 10^6^ cells/well in 300 μL (48-well plates) or at 2.4 × 10^5^ cells/well in 500 μL (24-well plates). Upon reaching confluency (usually following 24 h in culture), the astrocytes were again washed in PBS, and 300 or 500 μL serum-free X-Vivo 10 medium supplemented with penicillin, streptomycin, and l-glutamine was added to each well. Astrocytes were either left unstimulated or stimulated (Additional file 2: Figure S1) with a combination of IFNγ (10 ng/mL) and IL-1β (10 ng/mL) as previously reported [14]. After 24 h of incubation, the astrocytes were again washed thoroughly in PBS to minimize possible carry-over effects of activating cytokines, and fresh medium was added. Where indicated, astrocytes were either used in co-culture experiments with B cells (described below) or maintained in culture for an additional 24 h, with astrocyte-conditioned medium (ACM) then collected and stored at − 80 °C until use.

### B cell:astrocyte co-cultures

For direct B cell:astrocyte co-cultures, astrocytes were isolated as described above and plated at a density of 2.25 × 10^5^ cells in 300 μL per well in 48-well plates in DMEM supplemented with 10% FCS until they reached 80% confluence (2–3 days). DMEM was then removed and B cells, purified from the periphery of healthy donors, were directly co-cultured at a density of 3 × 10^5^ cells in 300 μL of X-Vivo 10. Survival responses were measured after 5 days of co-culture, while activation changes were assessed after 48 h of co-culture. For B cell:astrocyte transwell co-cultures, astrocytes were isolated and plated at a density of 2.4 × 10^5^ cells/well in 500 μL in 24-well plates and were either left unstimulated, or stimulated, as described above. BD Falcon cell-culture inserts (0.4 μm pore diameter) were then placed into the wells, and freshly isolated B cells were added to the upper compartments at a density of 2 × 10^5^ B cells in 200 μL serum-free X-Vivo 10 medium for the indicated duration, at which time the B cells were collected from the upper well and survival and activation were measured. For experiments assessing the effects of astrocytes on B cell subsets, the sorted B cell subsets were exposed in transwell to astrocytes, as described above, for 40 h. For experiments utilizing ACM, B cells were plated at a density of 1.5 × 10^5^ cells in 150-μL serum-free X-Vivo 10 medium per well in U-bottom 96-well plates, and 50 μL of ACM (or control medium, as indicated) was added to each well (representing 25% of the final volume). For functional blocking experiments, neutralizing antibodies to human IL-6 (clone 6708) human IL-15 (clone 34559) and BAFF (clone 148725), as well as appropriate isotype controls (all R&D Systems), were incubated at a final concentration of 1 μg/ml with ACM for at least 20 min prior to addition to the B cell cultures. At the end of all cultures, B cell survival and expression of the T cell co-stimulatory molecule CD86 were assessed by flow cytometry, as described below.

### T cell isolation and allogeneic stimulation

To determine whether astrocyte exposure can modulate the capacity of B cells to induce T cell responses, B cells that were pre-exposed to astrocyte-soluble factors in the transwell system described above were washed and co-cultured with T cells isolated from different healthy donors. Following PBMC separation, CD4+ T cells were isolated by positive selection using standard MACS separation (Miltenyi Biotec). T cell purity was confirmed by flow cytometry (routinely > 98%), and the T cells were then washed, resuspended in serum-free X-Vivo 15 medium, and stained with CFSE (20 μL CFSE 1% per mL). Allogeneic B cells that were previously cultured for 48 h under the different conditions described above were added to the freshly isolated CD4+ T cells in 300 μL serum-free X-Vivo 15 medium in 48-well plates at a B cell:T cell ratio of 1:4 (0.75 × 10^5^ B cells per 0.3 × 10^6^ T cells). T cells were also cultured alone, as a negative control, or with 2 μg/mL phytohemagglutinin (PHA, Sigma), as a positive control for T cell proliferation. Following 6 days in culture, the T cells were harvested and analyzed by flow cytometry as described below.

### Flow cytometry for T cell and B cell responses

Depending on the assay, B cell survival was assessed following 2, 3, or 5 days in culture by co-staining for CD20 (anti-CD20, 2H7, BD Bioscience), 7AAD (BD Bioscience), and Annexin V (BD Bioscience), with forward- (FSC) and side-scatter (SSC) properties also considered. Surviving B cells we considered to be Annexin V^−^/7AAD^−^ CD20^+^. B cell activation was assessed by quantifying upregulation of surface expression of the co-stimulatory molecule CD86 (anti-CD86, FUN-1, BD Bioscience). Following 2 days in culture, B cells were collected and washed using PBS containing 5% FCS, incubated with CD20 and CD86 antibodies for 20 min at 4°C, washed again and stained with Annexin V and 7AAD for 10 min at room temperature. T cell proliferation was quantified based on CFSE dilution following 6 days of culture as described above. All FACS acquisition was done using either FACSCalibur or LSRFortessa (BD Biosciences), and data were analyzed using FlowJo flow-cytometry10 analysis software (TreeStar, OR, USA).

### Statistical analyses

GraphPad Prism (versions 7 and 8) was used for all statistical analyses. Student’s unpaired *t* tests were used for statistical comparisons between two groups when the assumption of normal distribution was deemed appropriate. One-way ANOVA was used to compare across groups or conditions, and two-way ANOVA was used to compare several groups across different conditions.

## Results

### Human astrocytes support B cell survival and increase their co-stimulatory molecule expression

**Fig. 1:**
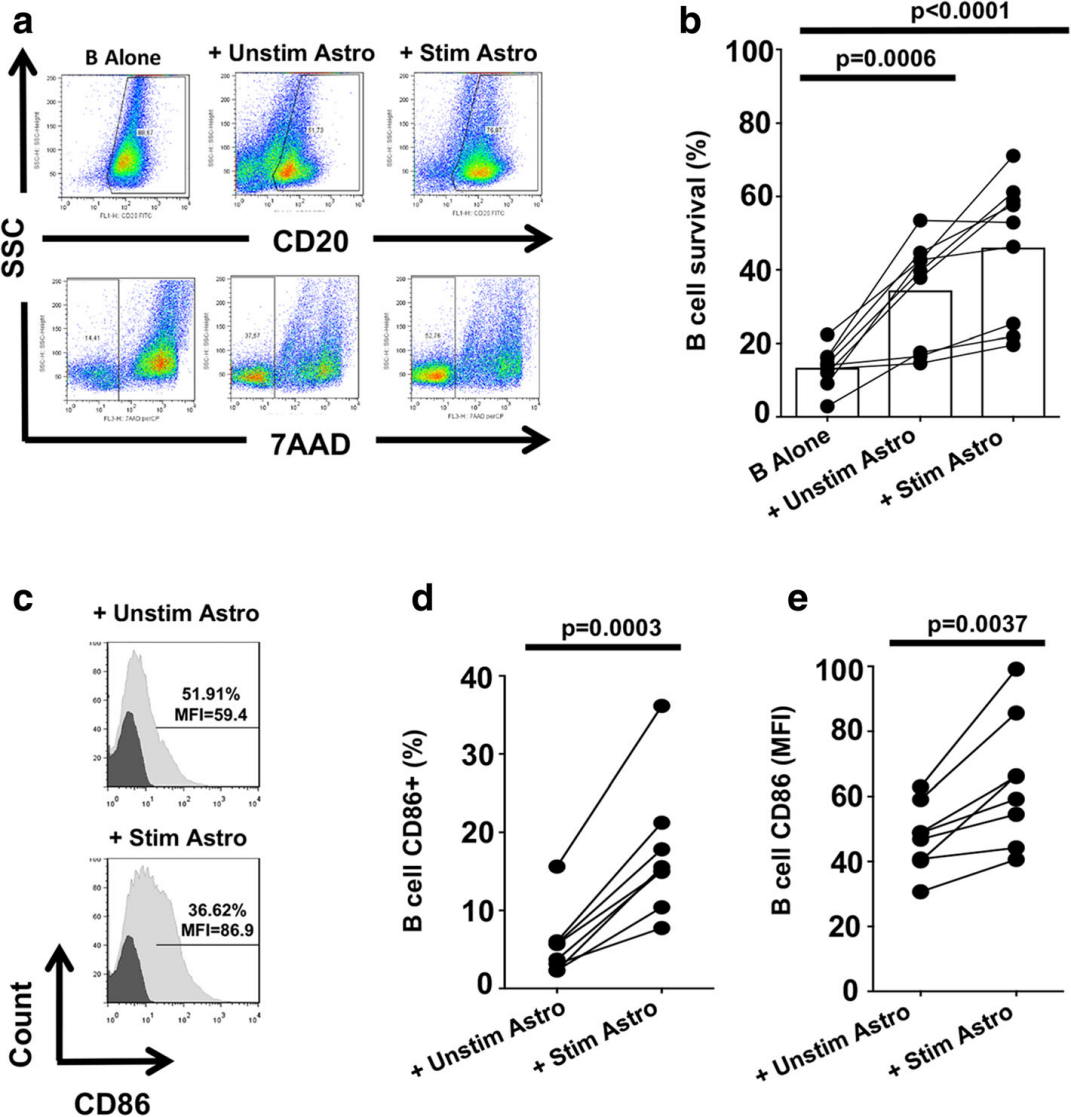
Human astrocyte co-culture increases B cell survival and CD86 expression. Human astrocytes (Astro) were stimulated (Stim) with recombinant human IFNγ (10 ng/mL) and IL-1β (10 ng/mL) or received no stimulation (Unstim) for 24 h. Astrocytes were then washed and co-cultured with human B cells in serum-free medium. B cells were also cultured without astrocytes in serum-free medium only (B alone). **a** Following 5 days of co-culture, we gated on CD20+ cells and assessed B cell viability through 7AAD staining. **b** Percentage of viable B cells from 9 independent donors after 5 days of co-culture. **c** CD86 expression in B cells was assessed by flow cytometry after 2 days. Percentage of CD86-positive cells (**d**) and CD86 MFI (**e**) from 8 independent donors (data were analyzed with one-way ANOVA (**b**) and paired *t* test (**c**); *p < 0.05; **p < 0.01; ***p < 0.001)

While human B cells cultured alone survived poorly (as expected), survival of B cells co-cultured with human astrocytes was significantly enhanced (Fig. 1a, representative donor; Fig. 1b, summary, *n* = 9), whether the astrocytes were previously cultured under basal conditions (*p* < 0.001, *n* = 9), or pre-activated with IFNγ and IL-1β (*p* < 0.001, *n* = 9) as a proxy for astrocytes pre-activated by an inflammatory environment. While the enhancement of B cell survival mediated by exposure to astrocytes was similar whether the astrocytes had been pre-activated or not (Fig. 1b), B cells exposed to pre-activated astrocytes also exhibited significant upregulation of the T cell co-stimulatory molecule CD86 (B7.2), as indicated by the percentage of B cells expressing CD86 (Fig. 1c; *p* < 0.001, *n* = 8) and their CD86 mean fluorescence intensity (MFI) (Fig. 1d; *p* < 0.001, *n* = 8).

### The effects of astrocytes on B cells can be mediated by astrocyte-secreted products

**Fig. 2:**
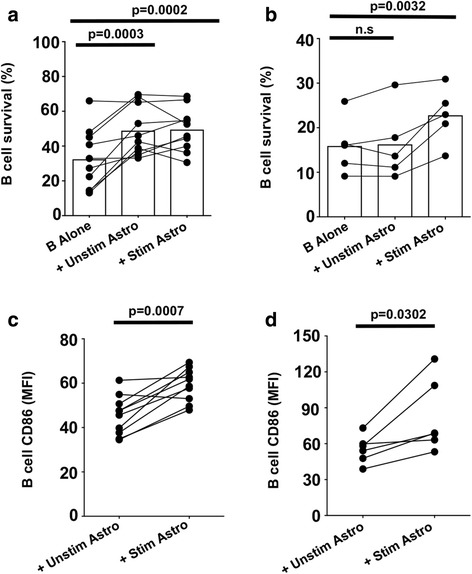
Soluble factors from human astrocytes increase B-cell survival and CD86 expression. (**a**, **c**) B cells were cultured in transwells in serum-free medium, either alone (B alone) or with either stimulated (Stim) or unstimulated (Unstim) human astrocytes (Astro). (**b**, **d**) B cells were cultured with 25% serum-free medium, which had not been conditioned by astrocytes (B alone), or with 25% astrocyte-conditioned medium (ACM) from stimulated (Stim) or unstimulated (Unstim) astrocytes. B-cell viability (**a**, **b**) was assessed after 2 and 3 days of co-culture using 7AAD and Annexin V staining. Data are shown from 9 independent experiments using different healthy-control donors. B-cell CD86 expression (**c**, **d**; MFI) was determined by flow cytometry following 2 days in culture. Data are shown for 5 independent experiments (one way ANOVA (2**a**, **b**), paired t-test (2**c**, **d**); n.s.: not significant; *: *p* < 0.05; **: *p* < 0.01; ***: *p* < 0.001)

To determine whether the effects of astrocytes on B cells are contact-dependent or can be mediated by astrocyte-soluble factors, we used two approaches: (i) co-culture of astrocytes and B cells in transwells, in which cells are not in direct contact but soluble factors can cross the 0.4-μm pore-size membrane bi-directionally; or (ii) addition of astrocyte-conditioned medium (ACM) to the B cell cultures for 3 days. As previously observed with the direct co-culture described above, incubation of B cells across a transwell from astrocytes also significantly enhanced B cell survival (Fig. 2a, *p* < 0.0001, *n* = 10), whether B cells were cultured in transwell with stimulated (*p* = 0.0002) or unstimulated astrocytes (*p* = 0.0003). ACM from pre-stimulated astrocytes also significantly increased B cell survival (Fig. 2b; *p* = 0.0032), while ACM derived from unstimulated astrocytes did not (Fig. 2b; *p* > 0.99). Compared to unstimulated astrocytes, stimulated astrocytes in transwell also significantly increased B cell surface expression of CD86 (Fig. 2c; *p* = 0.0007, *n* = 10), which was also observed with astrocyte-conditioned medium. Compared to ACM from unstimulated astrocytes, ACM from stimulated astrocytes was able to significantly increase B cell expression of CD86 (Fig. 2d; *p* = 0.032, *n* = 5). In a series of blocking experiments, neutralizing antibodies to either IL-6, IL-15, or BAFF (compared to the appropriate isotype control) did not abrogate the effects of astrocyte-soluble products on B cell responses (Additional file 3: Figure S2).

### Secreted products of activated astrocytes enhance the ability of B cells to activate T cells

**Fig. 3:**
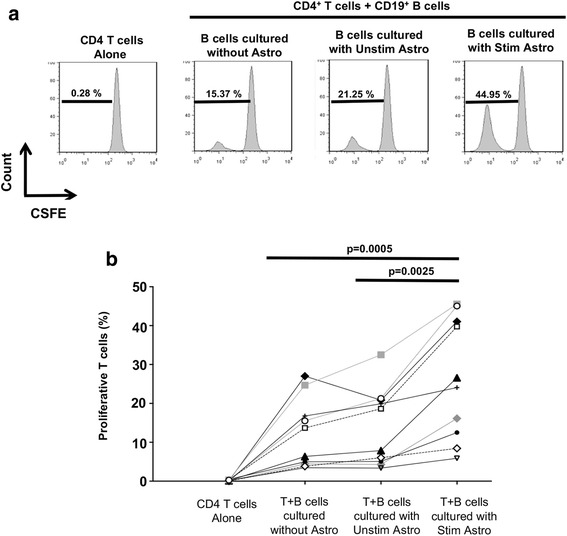
Astrocyte-exposed B cells enhance CD4+ T-cell responses. Human B cells were cultured in transwell as described above, either alone or with stimulated or unstimulated astrocytes. Following 2 days in culture, B cells were harvested, thoroughly washed and co-cultured with human T cells from allogeneic donors for 6 days at a B cell:T cell ratio of 1:4. T cell proliferation was assessed by flow cytometry using CFSE staining. **a** Representative data from a single donor. **b** Each line/symbol represents data from *n* = 10 independent experiments using different donors. (paired *t* test; **p < 0.01; ***p < 0.001)

Based on the observations above, we predicted that B cells pre-exposed to stimulated astrocytes might exhibit an enhanced capacity to activate T cells. As shown in Fig. 3 (*n* = 10), T cells cultured alone (negative control) did not proliferate. The addition of allogeneic B cells that were not pre-exposed to astrocytes induced an expected degree of allogeneic T cell proliferation (Fig. 3a, summarized in Fig. 3b). B cells that were pre-exposed in transwell to unstimulated astrocytes did not substantially enhance T cell proliferation; however, pre-exposure of B cells to stimulated astrocytes in transwell resulted in B cells that induced significantly greater T cell proliferative responses (Fig. 3b) and T cell secretion of IFNγ (Additional file 4: Figure S3).

### Astrocyte-secreted factors impact responses of MS-patient-derived B-cell subsets

**Fig. 4:**
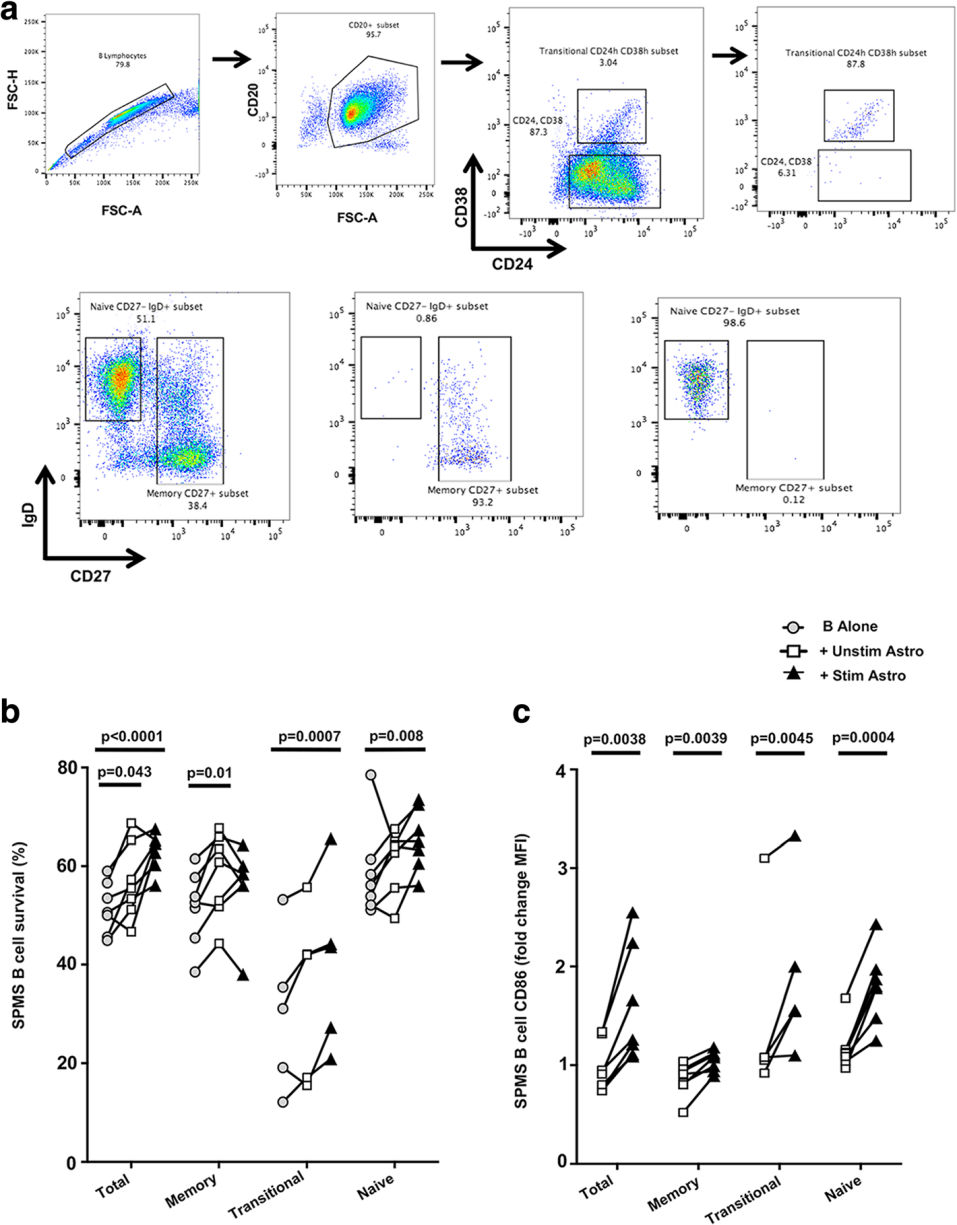
Astrocyte-secreted factors impact responses of MS-relevant B cell subsets. **a** B cells derived from untreated SPMS patients were sorted into total CD20+ B cells, naive CD20^+^ CD27^−^ IgD^+^ B cells, transitional CD20^+^ CD24^high^, CD38^high^, and memory CD20^+^ CD27^+^ IgD^−/+^ B cells and were then cultured either alone or with stimulated or unstimulated astrocytes in transwell, in serum-free medium X-Vivo 10 (*n* = 7). **b** B cell viability was assessed after 40 h of transwell co-culture using 7AAD and Annexin V staining; **c** CD86 MFI was determined by flow cytometry following 40 h in transwell co-culture. (two-way ANOVA test and paired *t* test; n.s. not significant; *p < 0.05; **p < 0.01; ***p < 0.001)

To establish whether the ability of astrocytes to support B cell survival and activation is recapitulated with MS-patient-derived B cells, we isolated B cells from untreated patients with secondary progressive MS (SPMS, Additional file 1: Table S1). Since the majority of B cells identified within the CNS of MS patients belong to the memory B cell pool [15–17], we further sorted the SPMS B cells into memory (CD27^+^ IgD^−/+^), naive (CD27^−^ IgD^+^), and transitional (CD24^high^ CD38^high^) B cell subsets (Fig. 4a) prior to exposing them to astrocyte-soluble factors. As shown in Fig. 4b, survival of total B cells derived from SPMS patients tended to be enhanced following exposure to non-activated (*p* = 0.043) and particularly to pre-activated (*p* < 0.0001) astrocytes. With some variability, astrocyte-derived soluble products supported survival of B cell subsets of SPMS patients. Though stimulated astrocytes did not decrease memory B cell survival (compared to the memory B cells alone), they did not appear to enhance B cell survival to the same extent as the unstimulated astrocytes did, possibly reflecting a process analogous to activation-induced cell death described in memory T cells. Soluble products of pre-activated astrocytes also induced substantial upregulation of surface expression of the T cell co-stimulatory molecule CD86 by the total B cells (*p* = 0.0038) and all B cell subsets (memory: *p* = 0.0039; transitional: *p* = 0.0045; and naive: *p* = 0.0004) (Fig. 4c).

## Discussion

Our study provides insights into cellular mechanisms by which B cells of MS patients may be fostered within an inflamed CNS environment, as well as into the potential for such B cells to contribute to the propagation of CNS-compartmentalized inflammation. The concept that B cells and plasma cells may be supported over time within the CNS of MS patients and hence might participate in ongoing disease activity has gained traction over the years (reviewed in [18]). Serial studies of cerebrospinal fluid (CSF) of patients have indicated that the same Ig oligoclonal banding (OCB) pattern of individual patients tends to persist over many years [10], suggesting that the same antibody-producing clones are maintained for extended periods. Follow-up studies quantifying B cell somatic hypermutation confirmed the long-term persistence and expansion of the same clones within the CSF of individual patients, and the same approach applied more recently to distinct tissue compartments within the CNS identified the same B cell and plasma-cell clones populating the CSF, parenchymal lesions as well as meningeal immune-cell collections of the same patients [17, 19–22]. This persistence of B cells within the chronically inflamed MS CNS is remarkable given the presence of several mechanisms that appear to normally limit responses to inflammation within the CNS. These include inflammation-mediated upregulation of expression of immune regulatory molecules such as HLA-G, CD200/CD200R, and SLAM5 by parenchymal CNS cells that can, in turn, downregulate responses of T cells and both resident microglia and infiltrating myeloid cells [23–25].

The potential significance of B cell persistence in the MS CNS, including within meningeal immune-cell collections, is highlighted by the growing recognition that B cells may contribute to disease-relevant immune responses through mechanisms that extend beyond antibody production (reviewed in [26]). In this regard, abnormalities in several antibody-independent functions of B cells have recently been described in patients with MS, including the demonstration of aberrant responses of MS B cells at the innate-adaptive interface, abnormal effector-cytokine profiles, over-expression of co-stimulatory molecules, and exaggerated activation of pro-inflammatory T cells and myeloid cells [11, 27–29]. While these abnormalities were all discovered in studies of B cells isolated from the periphery of patients, it would seem quite plausible that B cells within the CNS of these patients, which were populated from the periphery, may actively contribute to propagation of local inflammation and injury. Indeed, B cells of MS patients (unlike B cells from matched healthy controls) were recently found to mediate cytotoxicity to rodent oligodendrocytes as well as both human and rodent neurons in vitro through induction of apoptosis. This effect was mediated by B cell soluble products independent of antibody production [8, 9]. Shedding light on processes involved in supporting B cell persistence in the inflamed MS CNS and how such B cells, in turn, may contribute to local inflammation and injury is therefore of interest since these processes may represent attractive targets for novel therapies aimed at limiting or abrogating progressive disease.

Through a series of direct co-culture experiments, transwell experiments and use of human astrocyte-conditioned medium, we demonstrated that soluble factors form human astrocytes could substantially enhance survival of both healthy-control B cells and MS-patient-derived B cell subsets. Soluble products from astrocytes that were pre-exposed to inflammatory molecules (known to be present in the inflamed MS CNS) could also enhance B cell activation and their subsequent ability to more efficiently activate T cells. The observation that these effects were mediated through astrocyte-secreted factors and do not require direct astrocyte:B cell contact is noteworthy, since the pathology that appears most relevant to progressive MS is the subpial cortical pathology, which—while notable for demyelination, neuronal loss, microglial activation, and astrogliosis—exhibits very little, if any, infiltrating immune cells. Any role of immune cells, including B cells, is more likely to be mediated by immune cells within the meninges, which means that any interactions between glial cells and these immune cells would be predicated on actions of soluble molecules.

We noted that B cells exposed to astrocytes in transwell cultures exhibited increased survival whether the astrocytes were pre-activated or not. In contrast, when using astrocyte-conditioned medium (ACM), only ACM derived from pre-activated astrocytes could enhance B cell survival. This points to a bi-directional interaction between B cells and astrocytes: while ACM from non-activated astrocytes is not sufficient to support B cell survival, the non-activated astrocytes that were exposed to B cells in transwell produced soluble factors that were able to support B cell survival. We also noted that while B cell survival was enhanced by supernatants of both unstimulated and pre-stimulated astrocytes, only the pre-activated astrocytes could also enhance co-stimulatory molecule expression by B cells. This suggests that the astrocyte-derived soluble factors responsible for enhancing B cell survival differ from those that increase B cell activation/co-stimulatory molecule expression and APC capacity.

Prior reports demonstrating that astrocytes in the MS brain express abnormally high levels of BAFF (a known survival factor for B cells and plasma cells) and that BAFF is overexpressed in the CSF of MS patients [6] provide indirect support for the hypothesis that glial cells in an inflammatory environment may contribute to a local B cell fostering environment. In EAE, soluble TACI-Ig (atacicept) which can bind BAFF-ameliorated disease [30] while in the treatment of patients with optic neuritis and MS, atacicept appeared to exacerbate disease [31, 32]. Our series of experiments directly assessed the impact of human astrocytes on human B cells. We opted to focus on primary human astrocytes (rather than astrocytic cell lines) and since isolating primary adult human astrocytes remains a challenge we utilized human fetal-derived astrocytes. This is important since while using these cells is the closest one can model in vitro human astrocyte:immune-cell interactions, there are likely to be relevant differences between fetal and adult astrocyte responses. Indeed, we have found that fetal human astrocytes do not secrete measurable levels of BAFF (data not shown) and BAFF neutralization did not abrogate the effects of the astrocyte supernatants on B cells (Additional file 3: Figure S2e, f), indicating human fetal astrocytes are able to support B cell survival (and activation) through a BAFF-independent mechanism. The astrocyte-mediated enhancement of B cell survival and activation was also not impacted with neutralization of IL-6 or IL-15 in the astrocyte supernatants (Additional file 3: Figure S2a-d). Future experiments will be aimed at elucidating the distinct molecular mechanisms that underlie the ability of astrocytes to support B cell survival, and the ability of activated astrocytes to enhance B cell activation and subsequent T cell activation, which may prove particularly relevant to propagating CNS-compartmentalized inflammation and progressive MS.

Given our findings that astrocytes can support survival and activation of human B cells, and given prior work indicating that B cells of MS patients exhibit abnormal immune responses as well as the capacity to injure CNS cells, it was important to demonstrate that these astrocyte-mediated effects would also apply to MS-patient-derived B cells. We could document that B cells derived from untreated patients with progressive MS also exhibited enhanced survival and activation upon exposure to astrocyte-soluble products and, furthermore, that this was true for all major B cell subsets, including patient-derived memory B cells, which comprise the great majority of B cells found within the MS CNS [15–17]. Since there is growing recognition that progressive MS disease mechanisms are likely initiated well before patients manifest with clinical disease progression, we were curious to see how B cells of MS patients earlier in their disease course would respond and found that survival and activation of B cells derived from untreated patients with relapsing remitting MS were also enhanced following the same astrocyte exposure (Additional file 5: Figure S4).

It is now appreciated that activated astrocytes may exhibit distinct functional response profiles that may depend on how they are activated (Additional file 2: Figure S1). While our in vitro activation of astrocytes (using brief exposure to pro-inflammatory cytokines) may be more likely to induce pro-inflammatory A1 astrocytes with neurotoxic potential [33], it is of interest to consider how alternatively activated (A2) astrocytes that can exhibit neuroprotective properties [34–36] may differently influence CNS-compartmentalized B cells, and whether therapies aimed at modulating astrocytes from A1 to A2 responses may in turn also limit the potential contribution of B cells to pro-inflammatory cascades and CNS injury within the CNS.

## Conclusions

Our findings shed some light on human glial:immune interactions that may support survival of B cells within the inflamed CNS and their potential to propagate CNS-compartmentalized disease processes in MS. Future definition of molecular mechanisms underlying these interactions could contribute to novel therapeutic strategies aimed at targeting progressive disease, a major unmet need in the field of MS.

## Additional files

Additional file 1: **Table S1.** (DOCX 68 kb)

Additional file 2: **Figure S1.** *Confirming activation of human astrocytes.* Astrocytes were cultured for 24 h and were either left unstimulated or were stimulated with IFNγ (10 ng/ml) and IL-1β (10 ng/ml). After 24 h, the astrocytes were washed thoroughly and fresh medium was added. After an additional 24 h in culture, at which time cultures were imaged and supernatants were collected for subsequent measurement of astrocyte-secreted IL-6 by ELISA. Compared to unstimulated astrocytes (a), stimulated astrocytes exhibited activated morphology (b) and significantly-enhanced production of IL-6 (c; *p* = 0.0016; paired t-test). (TIFF 3951 kb)

Additional file 3: **Figure S2.** *Effects of astrocytes cytokine neutralization on B cell survival and activation.* B cells from HC were either cultured alone, or with stimulated astrocyte conditioned-medium (ACM), or with ACM pre-treated with neutralizing antibodies to IL-6 (a, b; anti-IL6: aIL-6), IL-15 (c, d; anti-IL-15: aIL-15) or BAFF (e, f; anti-BAFF: aBAFF); or pre-treated with corresponding isotype control antibodies. After 2 days of culture B cell viability was assessed using ANNEXIN V and 7AAD staining, and CD86 expression was measured by flow cytometry (representative experiment). (TIFF 4226 kb)

Additional file 4: **Figure S3.** *IFNγ production by proliferative T-cells.* Human B cells were cultured in transwell as described previously, either alone or with stimulated or unstimulated astrocytes. Following 2 days in culture, B cells were harvested, thoroughly washed and co-cultured with human T cells from allogeneic donors at a B-cell:T-cell ratio of 1:4. Conditioned media of B-cell:T-cell co-culure was collected and IFNγ was measured using ELISA (representative experiment). (TIFF 7670 kb)

Additional file 5: **Figure S4.** *Astrocyte-secreted factors also support survival and activation of relapsing remitting MS (RRMS) B cells.* B cells derived from patients with RRMS were cultured in transwell either with unstimulated human astrocytes or with astrocytes that had previously been stimulated as described above. (a) B-cell viability was assessed after 48 h of transwell co-culture using 7AAD and Annexin V staining; (b) CD86 MFI was determined by flow cytometry following 48 h of transwell co-culture. Data were analyzed using one way ANOVA test (*n* = 6 independent experiments; n.s.: not significant; *: *p* < 0.05; **: *p* < 0.01; ***: *p* < 0.001). (TIFF 2647 kb)

## Abbreviations

CNS: Central nervous system
CSF: Cerebrospinal fluid
HC: Healthy control
HFA: Human fetal astrocytes
MS: Multiple sclerosis
PMS: Progressive multiple sclerosis
RRMS: Relapsing remitting multiple sclerosis
