# Supplementary material for: Human central nervous system astrocytes support survival and activation of B cells: implications for MS pathogenesis
Source: J Neuroinflammation. 2018 Apr 19;15:114. doi: 10.1186/s12974-018-1136-2 (PMC5907187; doi:10.1186/s12974-018-1136-2)
Supplement: Supplementary file 1 — Table S1. (DOCX 68 kb) [file 12974_2018_1136_MOESM1_ESM.docx]

**Table S1**

| **MS Patients** | **Age** | **Sex** | **Diagnosis** | **Treatment Status** | **Steroids within 30 days** | **Disease duration** |
| --- | --- | --- | --- | --- | --- | --- |
| RRMS 1 | 23 | F | RRMS | Untreated | No | 5 |
| RRMS 2 | 24 | M | RRMS | Untreated | No | 1 |
| RRMS 3 | 43 | F | RRMS | Untreated | No | 11 |
| RRMS 4 | 37 | M | RRMS | Untreated | No | 1 |
| RRMS 5 | 50 | F | RRMS | Untreated | No | 10 |
| RRMS 6 | 43 | F | RRMS | Untreated | No | 1 |
| SPMS 1 | 56 | F | SPMS | Untreated | No | 14 |
| SPMS 2 | 72 | F | SPMS | Untreated | No | 12 |
| SPMS 3 | 55 | F | SPMS | Untreated | No | 24 |
| SPMS 4 | 72 | M | SPMS | Untreated | No | 5 |
| SPMS 5 | 71 | M | SPMS | Untreated | No | 14 |
| SPMS 6 | 60 | F | SPMS | Untreated | No | 13 |
| SPMS 7 | 68 | F | SPMS | Untreated | No | 12 |
