# Supplementary figures and images for: Human central nervous system astrocytes support survival and activation of B cells: implications for MS pathogenesis
Source: J Neuroinflammation. 2018 Apr 19;15:114. doi: 10.1186/s12974-018-1136-2 (PMC5907187; doi:10.1186/s12974-018-1136-2)

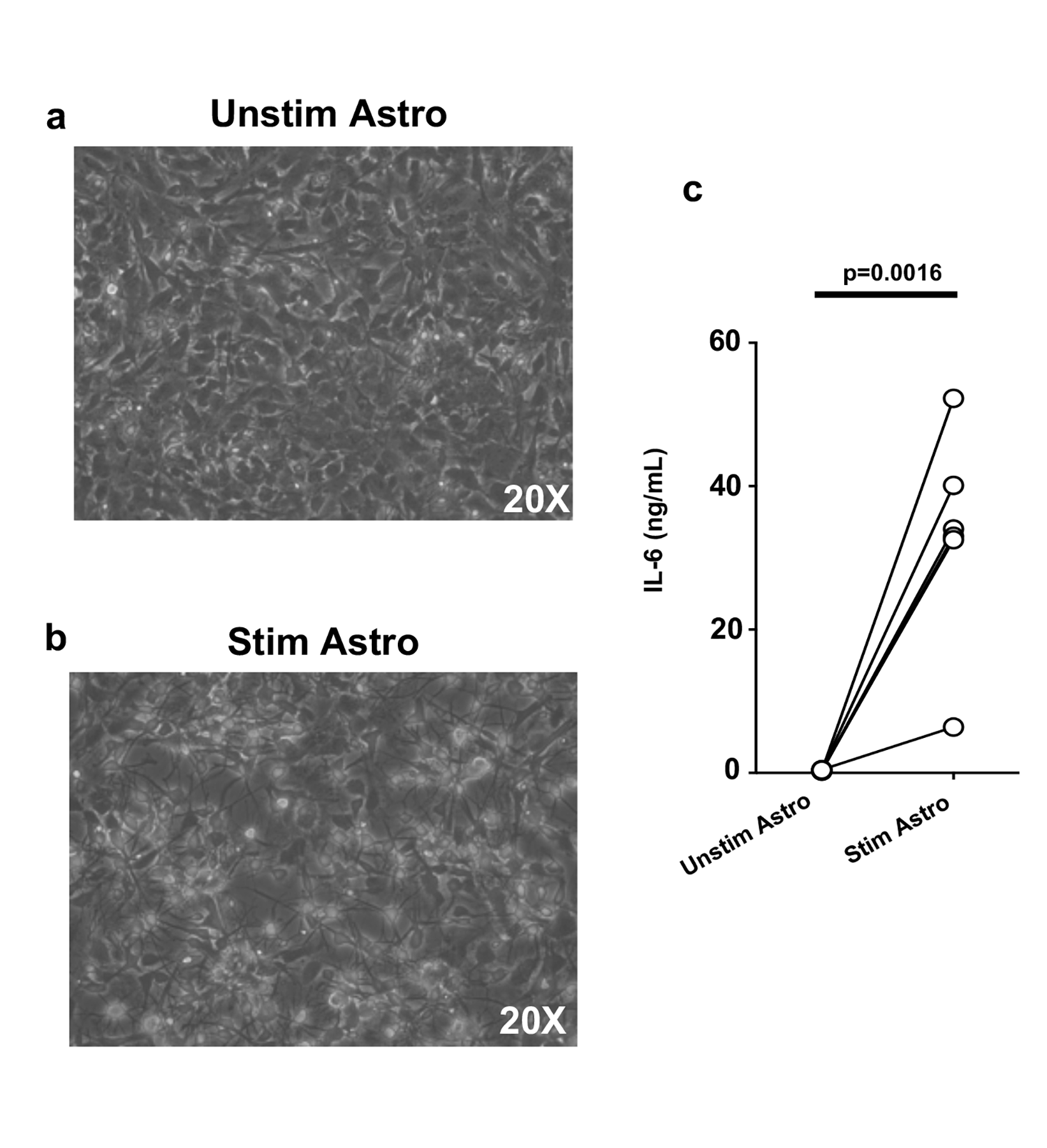

Supplement: Supplementary file 2 — Figure S1. Confirming activation of human astrocytes. Astrocytes were cultured for 24 h and were either left unstimulated or were stimulated with IFNγ (10 ng/ml) and IL-1β (10 ng/ml). After 24 h, the astrocytes were washed thoroughly and fresh medium was added. After an additional 24 h in culture, at which time cultures were imaged and supernatants were collected for subsequent measurement of astrocyte-secreted IL-6 by ELISA. Compared to unstimulated astrocytes (a), stimulated astrocytes exhibited activated morphology (b) and significantly-enhanced production of IL-6 (c; p = 0.0016; paired t-test). (TIFF 3951 kb) [file 12974_2018_1136_MOESM2_ESM.tif]

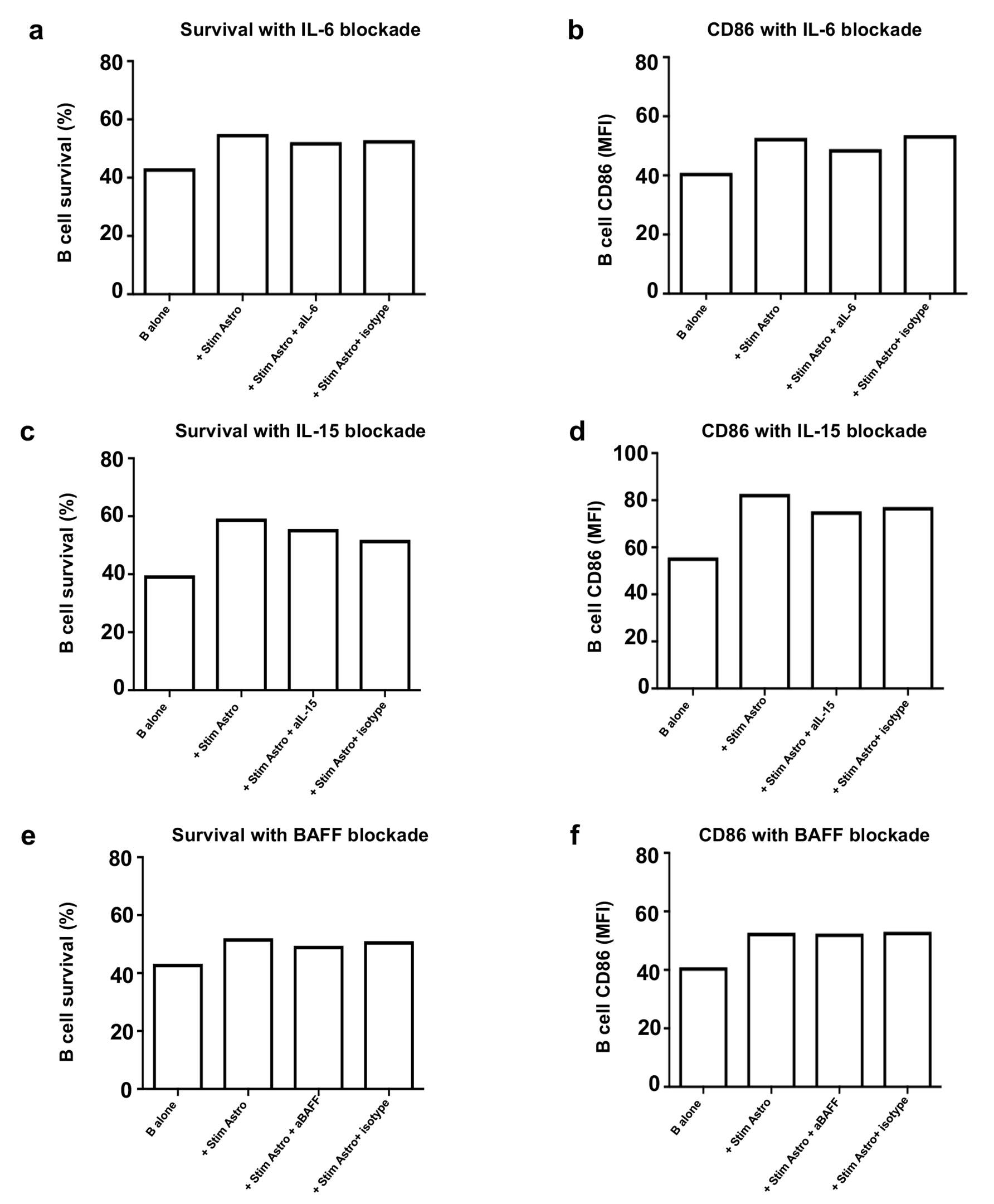

Supplement: Supplementary file 3 — Figure S2. Effects of astrocytes cytokine neutralization on B cell survival and activation. B cells from HC were either cultured alone, or with stimulated astrocyte conditioned-medium (ACM), or with ACM pre-treated with neutralizing antibodies to IL-6 (a, b; anti-IL6: aIL-6), IL-15 (c, d; anti-IL-15: aIL-15) or BAFF (e, f; anti-BAFF: aBAFF); or pre-treated with corresponding isotype control antibodies. After 2 days of culture B cell viability was assessed using ANNEXIN V and 7AAD staining, and CD86 expression was measured by flow cytometry (representative experiment). (TIFF 4226 kb) [file 12974_2018_1136_MOESM3_ESM.tif]

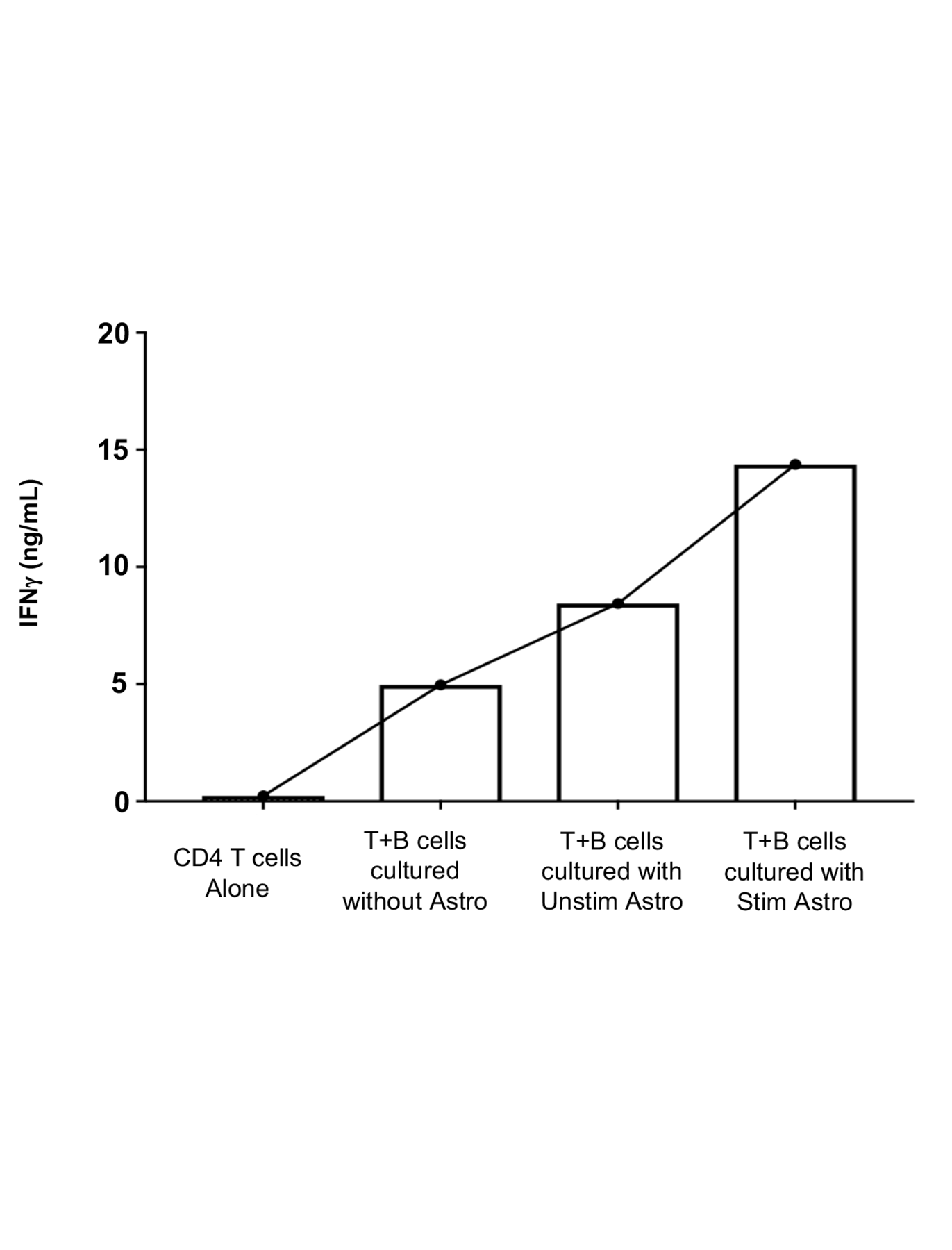

Supplement: Supplementary file 4 — Figure S3. IFNγ production by proliferative T-cells. Human B cells were cultured in transwell as described previously, either alone or with stimulated or unstimulated astrocytes. Following 2 days in culture, B cells were harvested, thoroughly washed and co-cultured with human T cells from allogeneic donors at a B-cell:T-cell ratio of 1:4. Conditioned media of B-cell:T-cell co-culure was collected and IFNγ was measured using ELISA (representative experiment). (TIFF 7670 kb) [file 12974_2018_1136_MOESM4_ESM.tif]

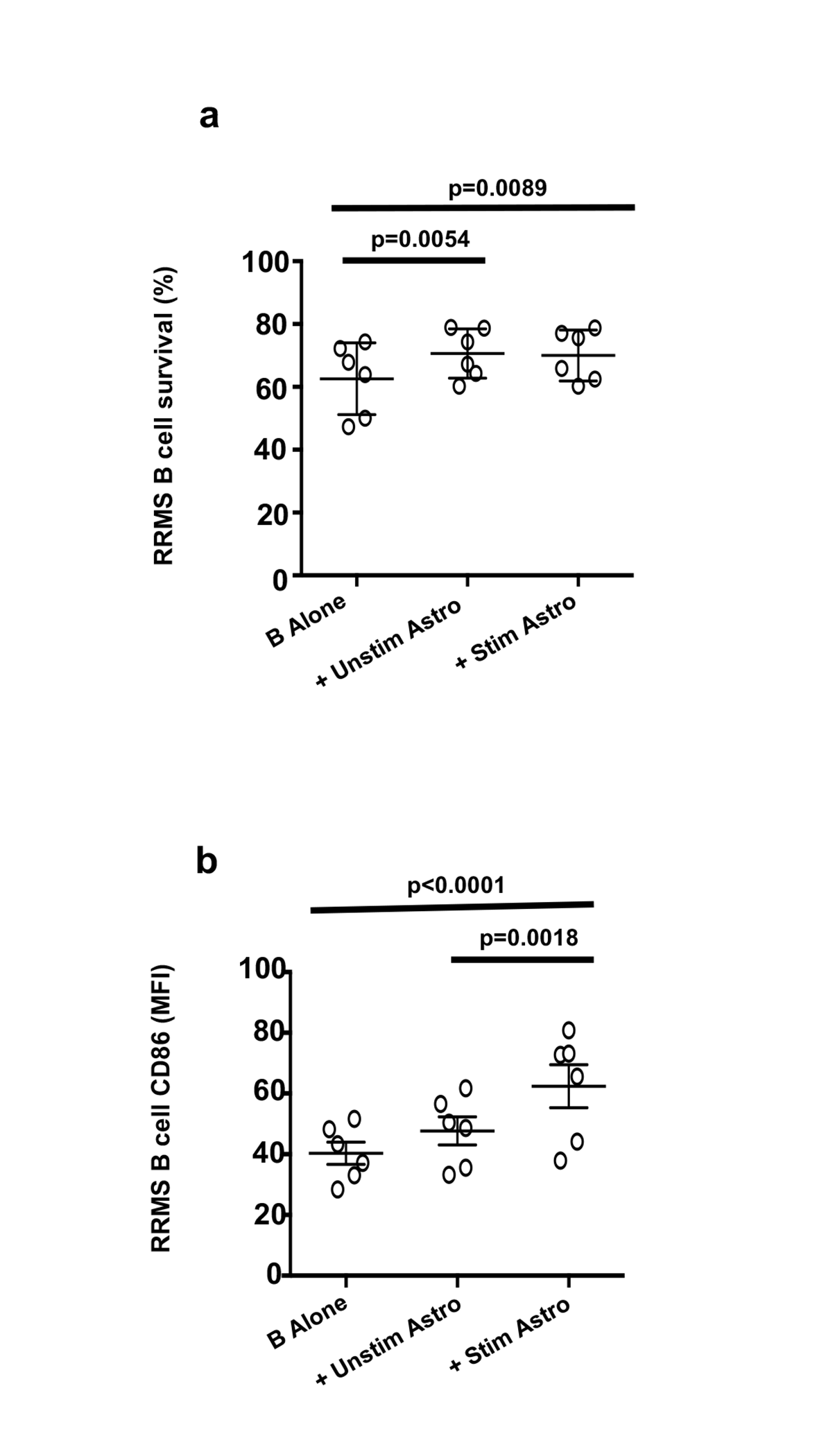

Supplement: Supplementary file 5 — Figure S4. Astrocyte-secreted factors also support survival and activation of relapsing remitting MS (RRMS) B cells. B cells derived from patients with RRMS were cultured in transwell either with unstimulated human astrocytes or with astrocytes that had previously been stimulated as described above. (a) B-cell viability was assessed after 48 h of transwell co-culture using 7AAD and Annexin V staining; (b) CD86 MFI was determined by flow cytometry following 48 h of transwell co-culture. Data were analyzed using one way ANOVA test (n = 6 independent experiments; n.s.: not significant; *: p < 0.05; **: p < 0.01; ***: p < 0.001). (TIFF 2647 kb) [file 12974_2018_1136_MOESM5_ESM.tif]
